# Change in Selected Mechanical Properties of Beech Wood at the Contact Drying

**DOI:** 10.3390/ma15217433

**Published:** 2022-10-23

**Authors:** Ivan Klement, Peter Vilkovský, Tatiana Vilkovská

**Affiliations:** Department of Wood Technology, Faculty of Wood Sciences and Technology, Technical University in Zvolen, T. G. Masaryka 24, 96001 Zvolen, Slovakia

**Keywords:** contact drying, beech, bending strength, Brinell’s hardness, impact toughness

## Abstract

The mechanical properties of wood have remarkable influence on its use in buildings. The improvement of the mechanical properties enables the reduction in the cross-sections of structural elements, particularly the increase in their load. The paper deals with the change in the mechanical properties of beech wood through the process of contact drying. Plate pressures of 1.0, 1.4, and 1.6 MPa at a temperature of 160 °C were used. It was found that contact drying increased the bending strength by more than 30% compared to convection drying. The value of Brinell’s hardness measured on the surface of the samples increased by 80 to 98% after contact drying, and the measured values of impact toughness were higher, about 31.1% compared to the samples dried by the convection drying method. As a result of contact drying, the density in the absolutely dry state increased for radial samples by 102.3 kg·m^−3^ and for tangential samples by 83.1 kg·m^−3^. The pressure of the plates also had an effect on the change in density.

## 1. Introduction

Beech wood (*Fagus sylvatica* L.) is widely represented in Europe and it has universal applications [1]. It is mainly used for furniture, plywood, decorative veneer manufacturing, or parquet (flooring). Most lumber must be dried prior to its use, since drying reduces shrinkage, increases strength, reduces weight, and improves overall manufacturing quality [2,3]. Timber or lumber is traditionally dried in kilns by processes often taking several weeks. Therefore, methods of drying wood at lower temperatures (120–180 °C) and shorter durations (1–5 h) were proposed to improve dimensional stability and mechanical properties, for example hardness or bending strength, with consideration for the wood applications and consumption during manufacturing [4]. Several ways to reduce the drying time from weeks to hours [5], for example contact drying, high-frequency drying, and so forth, are known in the world.

Contact drying is a method when wood is exposed to compression forces from heat-ed platens with the purpose of increasing the moisture loss and reducing warp [6]. It is usually dried by demonstrated schedules of a temperature and relative humidity specific for each tree species and lumber thickness [7]. In the case of deciduous tree species, the pressure from 0.4 up to 2.5 MPa is used. The temperature during contact drying should be in the range between 160 and 170 °C. Even higher temperatures were used in the research [8,9] on drying various deciduous tree species.

The process of drying results in more dimensionally stable wood in decreased humidity, and the mechanical properties show higher performance. The strength of beech wood is mainly due to its thick-walled fibrous cells. The variability in the structure of the fibrous cells of beech wood is the cause of the variability in its mechanical properties [10].

The study [10] was performed to investigate the effects of heat treatment on the mechanical properties (bending and compressive properties) under different heating conditions (180 °C for 12 h and 24 h, and at 210 °C for 3 h and 6 h). For the research two species of wood were used, pine (*Pinus densiflora*) and larch (*Larix kaempferi*), to assess the effects on bending properties in static and dynamic mode and compressive strength. The authors discovered that as the temperature and time were increased, bending properties of the heat-treated woods after heat treatment decreased. Modulus of rupture (MOR) values of red pine wood at heating conditions at 180 °C for 12 h and 24 h, and at 210 °C for 3 h and 6 h, decreased 30.83%, 45.49%, 46.85%, and 65.98%, respectively. In addition, the modulus of rupture (MOR) values of larch wood at the same conditions also decreased by 33.43%, 35.10%, 35.74%, and 61.75%, respectively. However, the compressive strengths of both species of wood that had been heat-treated at 180 °C for 12 h and 24 h, and at 210 °C for 3 h and 6 h, were greater than those of the control samples. Similarly, the study [11] presented the results of experimental studies on the influence of heat treatment on the mechanical properties of rowan (*Sorbus aucuparia* L.). Heat treatments were conducted at temperatures of 120, 150, and 180 °C for timespans ranging from 2 to 10 h. Results showed that treated samples had lower mechanical properties than those of the control samples. In this research, strength values of the samples decreased with increasing time and temperature of the treatments. The largest decrease was for impact bending strength (for example at temperature 120 °C from 116.572 MPa after 2 h to 113.785 MPa after 10 h), followed by tension strength parallel to grain, cleavage strength, compression strength parallel to grain, and tension strength perpendicular to grain when heat-treated at 180 °C for 10 h under the conditions stated. The modulus of elasticity in bending was stabilized, for example, at a temperature of 120 °C and 2 h drying with a value of 10,052.95 and at temperature of 150 °C and 2 h drying with a value of 9768.536 MPa. The research in [8] evaluated selected mechanical properties (toughness, hardness, and abrasion resistance) on eight hardwoods and one softwood. The authors concluded that the short time at elevated temperatures did not adversely affect the strength of the press-dried material. Similar research was conducted by [12], the purpose of which was to determine the effects of three drying method temperature/pressure combinations (1. Kiln drying at 116 °C, 2. Press drying at 172 or 345 kPa, and 3. Press drying at 177 or 210 °C) on mechanical properties. This study demonstrates that press drying does not necessarily improve the stiffness and strength properties. Press drying did not improve properties; neither did it result in substantial degradation. 

Other authors [13] researched the kinetics of vacuum contact drying of Jack pine (*Pinus banksiana*) wood boards (dimensions 50 × 100 × 2480 mm) under various drying temperatures and vacuum pressures. Drying temperatures and vacuum pressures ranged from 65 to 95 °C and from 0.016–0.05 MPa, respectively. The authors used three modes of vacuum-contact drying, presented as DS1, DS2, and DS3, in which parameters such as temperature and pressure were changed. Results indicated that the mechanical properties of dried samples were remarkably affected by drying temperature, pressure, and lumber grade. Mechanical test results were then compared to those for a conventional drying process, revealing that vacuum contact drying does not have a negative impact on the wood’s mechanical properties. However, one could not generalize this finding, taking into account, for example, the sample size. Authors looked the mechanical properties, for example, bending strength, modulus of elasticity in bending, etc. Bending strength was discovered at mode DS1 from 53.81 to 69.29 MPa, DS2 from 54.87 to 61.71 MPa, and DS3 from 52.78 to 63.85 MPa. The modulus of elasticity in bending was measured at modes DS1 from 8265.04 to 9768.69 MPa, DS2 from 7854.18 to 9461.39 MPa, and DS3 from 8153.27 to 9280.85 MPa. 

The objective of this research was to evaluate the effect of contact drying on the change in mechanical properties (bending strength, impact toughness, Brinell’s hardness) of beech wood with the use of different pressures: 1.0, 1.4, 1.8 MPa. The results showed that this is an innovative and fast process of drying for the improvement of selected mechanical properties of beech wood. The results were compared with convection hot-air drying.

## 2. Materials and Methods

Beech wood (*Fagus sylvatica* L.) was used for the experimental measurements. The samples were chosen from two beech logs with a diameter of 40 cm and a length of 300 cm. The samples were taken from the forest located in Môťová (475 m.a.s.l.) belonging to the University Forest Enterprise of the Technical University in Zvolen, Slovakia. 

**Figure 1:**
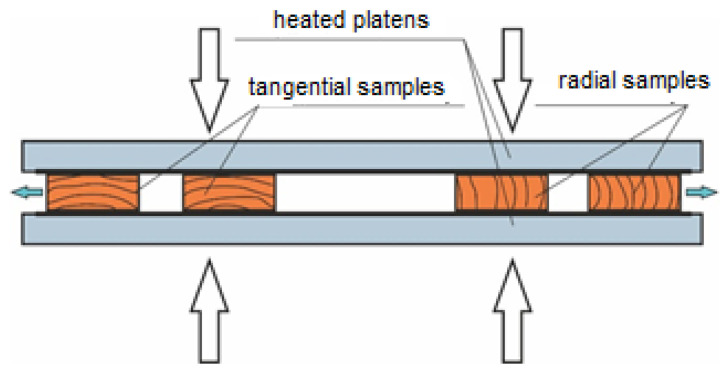
Scheme of sample storage during contact drying.

Radial and tangential samples were cut out (longitudinal and cross-section sawing) from the log, according to sawing patterns. Dimensions of drying samples were 120 × 800 × 30 mm (w × l × t). In total, 72 samples were investigated (36 radials and 36 tangential). The process of contact drying was conducted in hydraulic single-storied press type CBJ 500-5 (TOS RAKOVNIK, Rakovník, Czech Republic) at the Department of Wood Technology, Technical University in Zvolen, Slovakia (Figure 1). 

The temperature of the heating plates was 160 °C and three specific plate pressures of 1.0, 1.4, and 1.8 MPa were used. The drying parameters were chosen based on previous research as being optimal for beech wood. The group of samples was dried until the temperature measured in the centre of the samples reached the temperature of the pressing plate (t_p_– 5 °C) after insertion between the heating plates and reaching the specific pressure of the plates. The contact drying in the press was completed at that time. The temperature in the centre of the samples was measured by T-type thermocouples (Cu-CuNi), which were connected to a measuring unit Comet MS6R.

**Figure 2:**
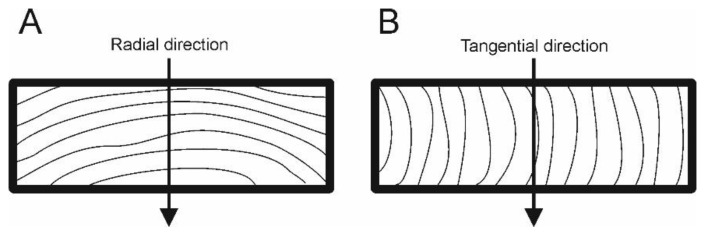
The direction of pressure of the plates in case of the radial (**A**) and tangential (**B**) samples.

One filling always consisted of samples from one radial and one tangential log (R and T). Figure 2 shows the radial and tangential samples and directions of effect pressure of the plates.

**Figure 3:**
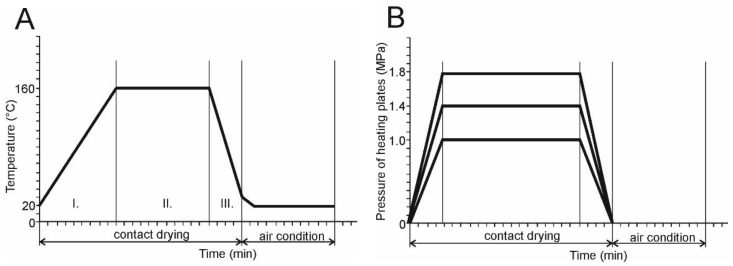
Temperature (**A**) and used pressures (**B**) during contact drying.

The drying regime is shown in Figure 3, where the course of temperatures and pressures in the individual stages of contact drying can be seen. The samples were dried at a constant temperature (II.) after gradual rise in temperature (I.) to 160 °C. The cooling phase was (III.) after reaching the desired temperature in the centre of the samples. The last phase was air conditioning at 20 °C. The air conditioning time was 24 h. The times of contact drying are given in Table 1 without the time of air conditioning.

Convection hot air drying was used for comparison. The drying regime was chosen for beech wood with a thickness of 30 mm. The maximum temperature of the drying environment was 65 °C. The process of drying was conducted in a laboratory kiln Memmert HCP 108 (Memmert GmbH + Co. KG, Schwabach, Germany). During this drying method, the moisture content of the samples was measured. Drying was completed when the samples had a moisture content of 8 ± 0.5%. The temperature in the wood was not measured.

Initial (*MC_i_*) and final (*MC_f_*) moisture content of wood were determined using the gravimetric method according to [14]. The moisture content was calculated using Equation (1):

(1) $$MC=\frac{m_{w}-m_{0}}{m_{0}}\;\cdot \;100\;\;\;\;\;(\%)\;$$

where *m_w_* is the weight of the wet sample (g) and *m*_0_ is the weight of the absolutely dry sample (g).

Density in oven-dried state was measured before and after drying. The measurement was performed under laboratory conditions. The density (*ρ*_0_) of wood at 0% moisture content was measured according to [15]. The oven-dried density was calculated using Equation (2):

(2) $$\rho_{0}=\frac{m_{0}}{V_{0}}\;\;\;\;\;(\mathrm{kg}\cdot m^{-3})$$

where *m*_0_ is the weight of oven-dried moisture samples (kg) and *V*_0_ is the volume of oven-dried moisture samples (m^−3^).

### 2.1. The Strength of Wood in Static Bending

**Figure 4:**
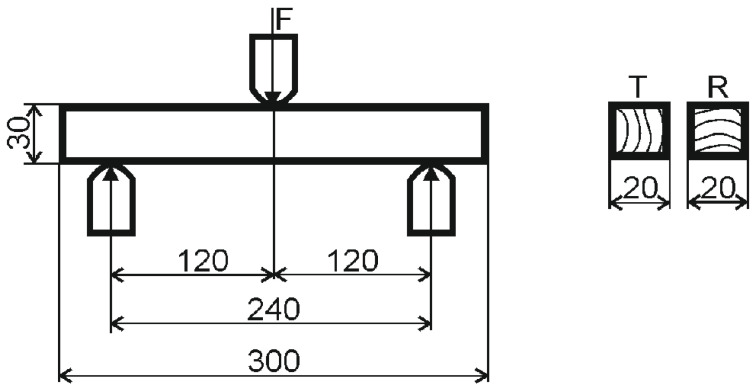
Test of wood in static bending.

The test of bending strength perpendicular to the fibres (Figure 4) and preparation of samples were conducted according to [16]. Samples with radial and tangential fibre direction were used for testing. 

The samples with dimensions of 30 × 20 × 300 mm were used for the static bending. The number of tree rings was >2 on the cross-section.

We tested the bending strength for both thicknesses on the IMAL IB600 device (Imal s.r.l. San Damaso Italy). 

Formula (3) was used with an accuracy of 1 MPa to calculate the bending strength of wood:

(3) $$\sigma_{ow}=\frac{3\;\cdot \;F_{max}\;\cdot \;l_{0}}{2\cdot b\;\cdot \;h^{2}}\;\;\;\;\;(\mathrm{MPa})\;$$

where *F_max_*—the maximum force (N), *l*_0_—distance of supports (mm), *h*—height of the sample relative to the supports (mm), *b*—width of sample (mm).

Moisture content was measured as needed after testing of static bending with an accuracy of 1 MPa according to Formula (4):

(4) $$\sigma_{o12}=\sigma_{ow}\;\cdot \;\left(1+\alpha \cdot \left(w-12\right)\right)\;\;\;\;\;(\mathrm{MPa})\;$$

where *σ*_*o*12_ is the bending strength at 12% moisture content, *α* is the correction coefficient of moisture, and *w* moisture content of wood (%) [17].

### 2.2. The Impact Toughness of Wood

The impact toughness in bending was measured using a Charpy hammer, according to the valid standard [18]. The samples were placed symmetrically on two supports 240 mm apart. The blow of the hammer was directed to the middle of the length of the sample. The consumed work was readied from the scale of the device with an accuracy of 1 J.

The toughness (*A*) was calculated according to Formula (5):

(5) $$A=\frac{Q}{b\;\cdot \;h}\;\;\;\;\;(J\cdot {\mathrm{cm}}^{-2})\;$$

where *Q* is break through work (J) and *b*, *h* are dimensions of cross section the sample (cm)

The impact toughness values were calculated to a humidity of 12% according to the formula

(6) $$A_{12}=A\left[1+\alpha \left(MC-12\right)\right]\;\;\;\;\;(J\cdot {\mathrm{cm}}^{-2})\;$$

where *A*_12_ is the impact toughness at a wood moisture content of 12%, *A* is measured impact toughness (J·cm^−2^), *α* is the correction coefficient (0.02), and *MC* is the moisture content of the samples (%).

### 2.3. Hardness of Wood

**Figure 5:**
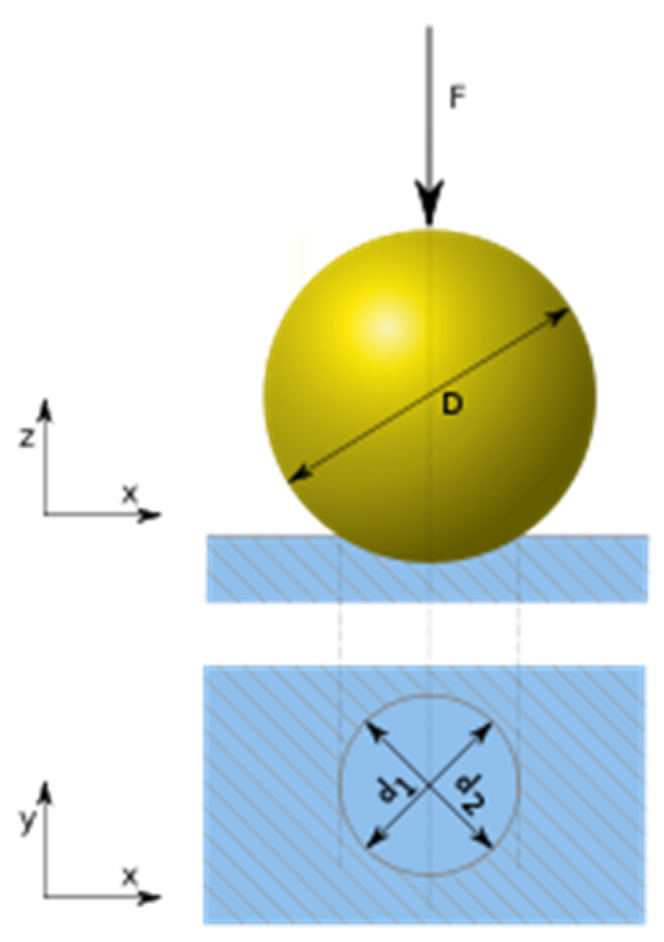
Measurement of Brinell’s hardness.

The static hardness of the samples was measured according to Brinell’s hardness. The surface hardness of the samples was measured at a depth of 1 and 2 mm below the surface. Hardness measurements at depths of 1 and 2 mm were performed after milling to the given thickness of the sample layer. Similarly, the surface hardness of the samples was also measured after they were dried by the convection drying method. The hardness test was performed according to [19]. A steel ball with a diameter of 10 mm (*D*) was pushed into the samples with a constant force of 1000 N. The time of the force was 15 s. The diameter of the imprinted area (*d*) was measured with a Brinell magnifier in two mutually perpendicular directions (Figure 5). 

The hardness according to Brinell was calculated by the formula

(7) $$HB=K\cdot \frac{2\cdot F}{\pi \cdot D\cdot \left(D-\sqrt{D^{2}-d^{2}}\right)}\;\;\;\;\;(\mathrm{MPa})\;$$

where *HB*—Brinell’s hardness (MPa), *K =* 1/*gn*, *gn*—gravitational acceleration, *F*—force acting on the steel ball (N), *D*—diameter of steel ball (mm), and *d*—diameter of the imprinted surface in wood (mm).

The hardness values were calculated at a moisture content humidity of 12% according to the formula

(8) $$HB_{12}=HB\left[1+\alpha \left(MC-12\right)\right]\;\;\;\;\;(\mathrm{MPa})\;$$

where *HB*_12_ is the Brinell’s hardness at the moisture content of 12%, *HB* is the measured Brinell’s hardness (MPa), *α* is the correction coefficient (0.025), and *MC* is the moisture content of samples (%).

## 3. Results and Discussion

The measured average values of moisture content, drying time, and density of the samples are shown in Table 1. The values are shown for individual pressures and types of samples. 

**Table 1:** Initial and final moisture content of the samples, drying time and density of the samples.

Type of Samples	Pressure of Plates (MPa)	Moisture Content *MC* (%)		Drying Time (min)	Density *ρ*_0_ (kg·m^−3^)	
		Initial	Final		Before Drying	After Drying
Radial	1.0	77.48	3.95	80	675.61	770.92
	1.4	80.27	6.08	80	684.16	785.15
	1.8	69.5	5.52	90	675.62	786.47
Tangential	1.0	71.87	5.78	100	669.93	722.07
	1.4	54.14	5.37	110	663.08	752.35
	1.8	55.54	4.87	90	666.95	774.85

Contact drying was conducted at a constant temperature of 160 °C. The process was controlled based on the temperature measurement in the centre of the samples. The average final moisture content was 5.26%. The drying time was short, which was due to the very intense transfer of heat to the dried samples and the removal of moisture through the fronts and sides of the samples. During convection drying, the average initial moisture content of the samples was 79.56% and the final moisture content was 8.28%. The drying time was 389 h.

Contact drying increased the density values in the absolutely dry state. Increases in density were discovered in the radial and tangential direction of samples (Table 1). Density after drying showed that the pressure of the plates can cause a change in density during contact drying. The greater increase in density was in the case of radial samples, which was caused by the pressure direction of the plates. The thickness of the samples was decreased from 3.34 to 5.38 mm after contact drying.

The average measured values of bending strength during contact drying and convection drying are shown in Table 2.

Contact drying increased the bending strength by an average of 34.3% compared to convection drying. It is a remarkable increase, while in the drying process there was an increase in density by an average of 14.1%. Similar results were discovered in the work [13]; their research was focused on the kinetics of vacuum contact drying of Jack pine (*Pinus banksiana*) at various drying temperatures and pressures (t = from 65 to 95 °C and vacuum pressure from 0.016–0.05 MPa). Results also indicated that the mechanical properties of dried samples were remarkably affected by drying temperature and pressure, among other things. Mechanical test results were then compared to those for a conventional drying process. They discovered that vacuum contact drying does not have a negative impact on the wood’s mechanical properties. The average measured values of bending strength during vacuum contact drying were 63.5 MPa and of convection drying were 70.66 MPa. Another study [10] focused on two species of wood, pine (*Pinus densiflora*) and larch (*Larix kaempferi*), which were used were to assess the effects of heat treatment on bending properties in static and dynamic mode and compressive strength. The authors discovered that as the temperature and time were increased, bending properties of the heat-treated woods after heat treatment decreased. However, the compressive strengths of both species of wood that had been heat-treated at 180 °C for 12 h and 24 h, and at 210 °C for 3 h and 6 h, were greater than those of the control samples. This was also shown by the study [11], which presented the results of experimental studies on the influence of heat treatment on the mechanical properties of rowan (*Sorbus aucuparia* L.). Results showed that treated samples had lower mechanical properties than those of the control samples. In this research, strength values of the samples decreased with increasing time and temperature of the treatments.

Table 3 and Table 4 show the statistical characteristics of the bending strength of beech samples dried by contact drying.

The differences in average values and other statistical characteristics of bending strength between radial and tangential samples were small (Table 3). The deviations in the measured samples were comparable in all the experiments.

In the mathematical analysis (Table 4), the statistically remarkable influence of plate pressure on the increase in bending strength was confirmed. When comparing radial and tangential samples, higher values of strength for tangential samples were measured. However, the difference was not statistically significant.

During impact toughness tests the average measured values during contact drying and convection drying were compared. The data are presented in Table 5.

Table 6 shows the statistical characteristics of the impact toughness of samples dried by contact drying. Table 7 shows the statistically remarkable effects of direction, annual rings, and plate pressure.

Similar to the bending strength is the increase in impact toughness for samples dried by the contact drying; they are higher by 31.1% compared to samples dried by the convection drying. Samples dried in this way have increased bending strength and are not brittle at the same time. The statistically significant influence of the plate pressure or the direction of the annual rings was not confirmed.

**Figure 6:**
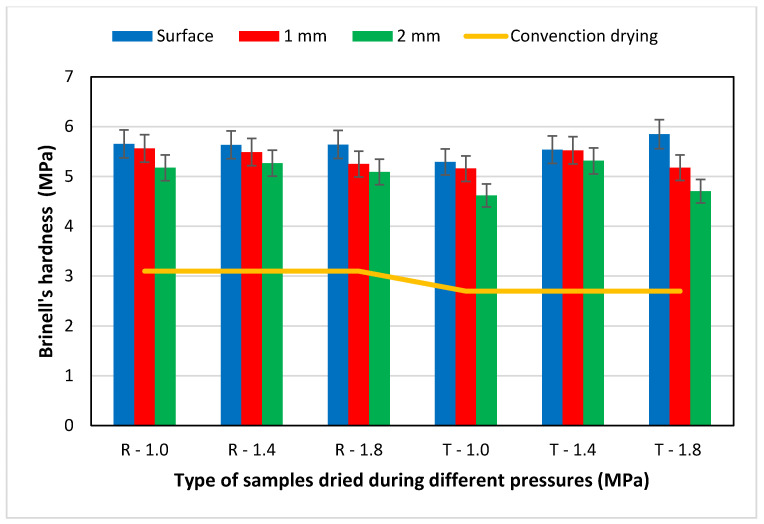
Brinell’s hardness values of beech samples after contact drying and convection drying on the surface, 1 and 2 mm below the surface.

The hardness of the surface layer of beech wood increased remarkably due to contact drying. The measured values of Brinell’s hardness for individual groups of samples during contact drying and convection drying are shown in Figure 6. The cited work [9] evaluated the mechanical properties of toughness, hardness, and abrasion resistance on eight hardwoods and one softwood. It was concluded that the short time at elevated temperatures did not adversely affect the strength of the contact drying material.

**Figure 7:**
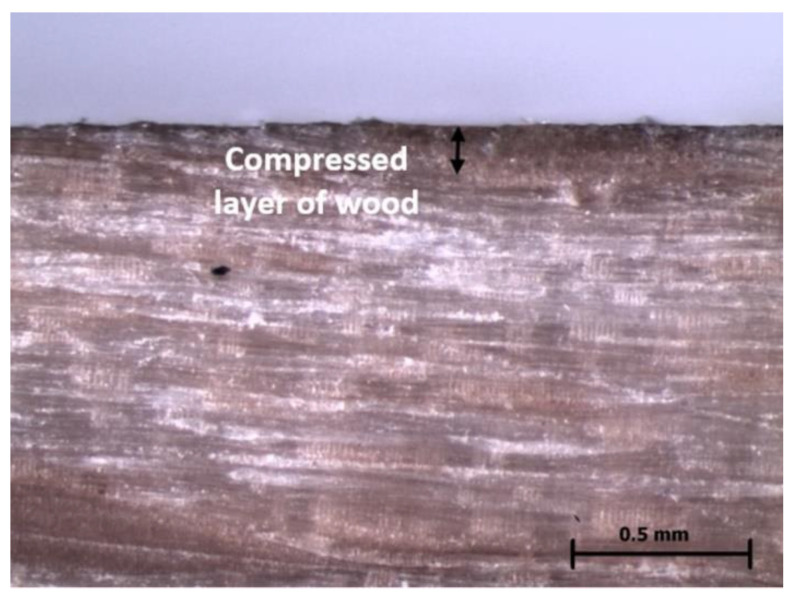
Microscopic view of the differently coloured surface layers of beech wood after contact drying at 60× magnification (type of microscope LEICA MZ 95).

During contact drying, surface hardness was increased by 80 to 98% compared to convection drying of samples. This difference was statistically remarkable. When we compare the hardness of the samples that were measured on their surface, the differences between radial and tangential samples are minimal. Different pressure of the plates had no remarkable effect on the surface hardness values. Hardness values decreased from the surface to the centre of the samples. A remarkable difference in hardness from the surface was in the tangential samples at a plate pressure of 1.8 MPa. There was a statistically remarkable difference in hardness for tangential samples at a plate pressure of 1.8 MPa between surface hardness and hardness at a depth of 2 mm. In addition, visible in Figure 7 is the colour difference between the very hard surface layer and the rest of the sample. The reasons for this colour difference were pressure and temperature during contact drying, which caused remarkable changes in the selected mechanical properties, for example hardness of surface. After convection, drying was not discovered to compress the surface of the wood (colour difference on the surface of samples).

## 4. Conclusions

The aim of the work was to determine how selected mechanical properties of beech wood change through the contact drying process. To determine the effect of the pressure of the heating plates and the inclination of the annual rings on the bending strength, impact toughness and hardness of the dried wood were studied. Beech samples with a thickness of 30 mm were used with radial and tangential course of annual rings. Drying was carried out at a temperature of heating plates of 160 °C and pressures of 1.0 MPa, 1.4 MPa, and 1.8 MPa. The results were compared with convection hot-air drying.

The following conclusions were stated from measured data:

The transfer of heat to the dried wood is very intense and contact drying times are remarkably shorter compared to convection drying.
Radial samples dried faster and the pressure of the heating plates did not affect the final drying time.
The density in the absolutely dry state as a result of contact drying increased for radial samples by 102.3 kg·m^−3^ and for tangential samples by 83.1 kg·m^−3^.
In addition to the inclination of the annual rings, the pressure of the plates also had an effect on the change in density.

Change of selected mechanical properties:

The increase in bending strength in the contact drying compared to convection drying was more than 30%. The pressure of the heating plates had a statistically remarkable effect on increasing the bending strength with increasing pressure.
The measured values of impact toughness were higher by 31.1% compared to the samples dried by the convection drying method.
The Brinell’s hardness value measured on the surface of the samples increased by 80 to 98% after contact drying compared to convection drying. Differences in surface hardness between radial and tangential samples were unremarkable and the influence of plate pressure was not confirmed either.
The hardness measured in the zones below the surface (1 and 2 mm) decreased and the decrease was more pronounced in samples dried at a plate pressure of 1.8 MPa as well as tangential samples.

The results showed a very favourable effect of contact drying on the change in the observed mechanical properties of beech wood. Increase the bending strength improves the use of wood in elements with increased load. Increase in the hardness of the surface after contact drying will allow the use of such dried wood in places with high stress, such as floors. In addition, of interest is the short drying times achieved and the possibility to partially change the final mechanical properties of the dried wood by changing the pressure of the heating plates.

## Figures and Tables

**Table 2:** Comparison of bending strength of samples after contact drying and convection drying.

Contact Drying	The Bending Strength (MPa)	
Pressure (MPa)	Radial	Tangential
1.0	121.86	125.47
1.4	126.43	132.13
1.8	131.67	139.07
**Convection drying**	96.36	

**Table 3:** Statistical characteristics during contact drying—bending strength.

Pressure (MPa)	Direction	Arithmetic Mean	Standard Error	95% Confidence Interval		Number of Measurements	Standard Deviation	Variation Coefficient
				Left Border	Right Border			
1.0	R	121.86	1.46	118.87	124.85	30	8.00	6.57
	T	125.47	1.32	122.77	128.17	30	7.24	5.77
1.4	R	126.43	2.76	120.78	132.08	30	15.13	11.96
	T	132.13	1.47	129.13	135.14	30	8.05	6.09
1.8	R	131.67	1.56	128.48	134.86	30	8.55	6.49
	T	139.07	1.86	135.27	142.87	30	10.18	7.32

**Table 4:** Two-way ANOVA—bending strength.

Source of Variability	Sum of Squares	Degrees of Freedom	Variance	F Test	p Level of Significance
Total average	7,952,095	1	7,952,095	118,435.6	0.000
Pressure	18,316	2	9158	136.4	0.000
Direction	4	1	4	0.1	0.816
Pressure—Direction	13,154	2	663	9.9	0.000

**Table 5:** Comparison of impact toughness of samples after contact drying and convection drying.

Contact Drying	Impact Toughness (J·cm^−2^)	
Pressure (MPa)	Radial	Tangential
1.0	8.61	10.43
1.4	8.74	11.46
1.8	8.88	10.44
**Convection drying**	7.77	7.12

**Table 6:** Statistical characteristics during contact drying—impact toughness.

Pressure (MPa)	Direction	Arithmetic Mean	Standard Error	95% Confidence Interval		Number of Measurements	Standard Deviation	Variation Coefficient
				Left Border	Right Border			
1.0	R	9.69	0.32	9.02	10.35	24	1.57	16.24
	T	9.93	0.21	9.48	10.37	24	1.05	10.60
1.4	R	10.39	0.40	9.56	11.23	24	1.98	19.04
	T	10.73	0.31	10.09	11.37	24	1.52	14.15
1.8	R	9.54	0.28	8.96	10.12	24	1.37	14.38
	T	9.96	0.25	9.45	10.47	24	1.21	12.11

**Table 7:** Two-way ANOVA—impact toughness.

Source of Variability	Sum of Squares	Degrees of Freedom	Variance	F Test	p Level of Significance
Total average	27,607.54	1	27,607.54	12,563.47	0.000
Pressure	8.79	2	4.4	2	0.137
Direction	3.23	1	3.23	1.47	0.226
Pressure—Direction	2.04	2	1.02	0.47	0.629
